# Anatomical Methods of Approach in Operation on the Long Bones of the Extremities

**Published:** 1919

**Authors:** 


***Anatomical Methods of Approach in Operation on the Long Bones of the Extremities.*** By JAMES E. THOMPSON, *Annals of Surgery,* September 1918.

In exposing the long bones, the following principles must be scrupulously observed :

(1) Easy access to the site of fracture or focus of disease. As far as possible we should try to avoid deep wounds where shallow ones will suffice. (2) Preservation of all nerves, both sensory and motor. Even cutaneous nerves should be preserved. (3) Prevention of unnecessary injury to muscles. As far as possible the approach should be between the muscles. In some instances, however, the muscles may be split parallel to their fibers without doing any permanent damage. (4) The preservation of the vascular supply. It is always wise to choose a route remote from the blood-vessels. Injury of the nutrient arteries of the bones must also be avoided.

The methods of approach depend entirely upon the anatomical stucture of the bones.

The distal end of the radius is accessible : (1) In front of the base of the styloid process by passing between the brachioradialis insertion and the radial artery, and exposing the bone to the lateral aspect of the pronator quadratus. (2) Above the base of the styloid process by passing between the tendon of the brachioradialis and the extensor carpi radialis longus. (3) Over a small area of the posterior surface just above the dorsal carpal ligament, between the extensor pollicis longus and the extensor pollicis brevis.

The shaft of the radius is accessible : (1) Along the lower third of its lateral border. (2) Along a narrow strip of the posterior surface situated between the insertion of the pronator teres and the origin of the abductor pollicis longus. The dissection passes superficially to the lateral side of the extensor digitorum communis, deeply between the abductor pollicis longus and the extensor carpi radialis brevis. (3) The upper third of the posterior surface can be reached by a prolongation upward of the incision in No. 2 as far as the external epicondyle.

The proximal end of the radius is accessible from behind by splitting the aponeurosis of origin of the extensor digitorum communis from the external epicondyle by a vertical cut.

The following are the safest methods of exposing the humerus :

A. *Shaft.* (1) In the lower third, the bone can be reached safely by the posterior route, the deep dissection splitting the triceps muscle parallel to its fibers. (2) In the middle third : (*a*) the safest is the anterior route which passes between the biceps and the insertion of the deltoid in its upper part, and along the outer edge of the biceps in its lower part. (*b*) The lower part of this area can be reached from behind by splitting the triceps. (3) In the upper third, the anterior route between the pectoralis major and the deltoid is the best and the safest.

B. *Articular Ends.* (1) Lower articular end : the lateral aspects of the condyles can be reached through small incisions directly over their prominent subcutaneous parts. To apply a plate properly, the best route is a posterior vertical incision which splits the triceps muscle near its insertion. (2) Upper articular end : an incision along the anterior margin of the deltoid gives the best exposure in most cases. If supplemented by a transverse cut backward through the deltoid muscle near its origin, the exposure is very complete.

*The Tibia.* (1) The route of choice is along the line of its subcutaneous surface from the medial tuberosity proximally to the tip of the medial malleolus distally. (2) Along the line of the medial border of the tendo Achillis and the flexor pollicis longus, to expose the posterior surface of the distal end of the shalt for tendon implantation and fixation. (3) Along the line of lateral border of the tibialis anterior, to expose the anterior surface of the distal end of the shaft for tendon implantation and fixation.

*The Fibula.* (1) Along the line of subcutaneous surface of the lower fourth of the shaft and the lateral malleolus. (2) Along the posterior peroneal septum for the upper three-quarters of the shaft.

*The Femur.* (1) Vertically upwards from either lateral or medial epicondyle for the lower epiphysis and the lower quarter of the shaft. (2) An antero-lateral incision lateral to the rectus femoris for a small area at the junction of the middle and lower thirds of the shaft. (3) An external incision for the upper three-quarters of the shaft along a line drawn from the tip of the throcanter major to the outer border of the patella. (4) Between the vastus lateralis in front, and the short head of the biceps cruris and the insertion of the gluteus maximus behind. (5) Along the line of a medial incision extending vertically upwards from the abductor muscle to expose the posterior surface of the lower fourth of the shaft. (6) The anterior oblique incision lateral to the line of the upper end of the sartorius muscle for the exposure of the hip-joint, the neck of the femur, and the upper part of the shaft.

